# One-carbon-derived bioactive peptides improve reproductive performance via regulating placental nutrient transport and offspring glycolipid metabolism

**DOI:** 10.1038/s41538-026-00769-9

**Published:** 2026-02-27

**Authors:** Lu-min Gao, Xu-dong Yang, Shu-fan Liu, Lu Liu, Xiao-fan Ma, Shu-guang Liu, Xin Wu

**Affiliations:** 1 State Key Laboratory of Engineering Biology for Low-Carbon Manufacturing, Tianjin Institute of Industrial Biotechnology, Chinese Academy of Sciences, Tianjin, China; 2 Beijing Chase Future Biotech Co. Ltd, Beijing, China

**Keywords:** Biochemistry, Biotechnology, Molecular biology, Physiology

## Abstract

This study aimed to develop novel bioactive peptides from *Pichia pastoris* (PpBP) as a potential functional ingredient for maternal nutrition. A high-efficiency strain was obtained through generated by ARTP mutagenesis of a winery by-product isolate and optimized via automated fermentation, yielding an enzymatic hydrolysate rich in short-chain peptides (Content = 30.73%). In vitro assays demonstrated that PpBP significantly upregulated the expression of intestinal peptide transporter PEPT1 in IPEC-J2 cells. Molecular docking revealed that dipeptides, especially Leu-Pro, can directly bind to the active site of PEPT1. Maternal PpBP (2 g/kg) supplementation from late gestation through lactation significantly reduced the incidence of IUGR and improved offspring growth performance. Mechanistic investigations indicated that PpBP intake modulated placental nutrient transport function, altering the expression of key glucose and lipid transporters and downregulating p38 MAPK and p-AKT signaling pathways. Placental transcriptomics further highlighted enriched pathways in Ras/Wnt signaling and lipid metabolism. In neonatal piglets, maternal PpBP supplementation shifted hepatic metabolism towards gluconeogenesis while suppressing glycolysis and TCA cycle activity. In conclusion, *P. pastoris*-derived bioactive peptides improved fetal growth and neonatal development by regulating maternal peptide absorption (via PEPT1 activation) and subsequently optimizing placental nutrient transport and fetal hepatic energy metabolism.

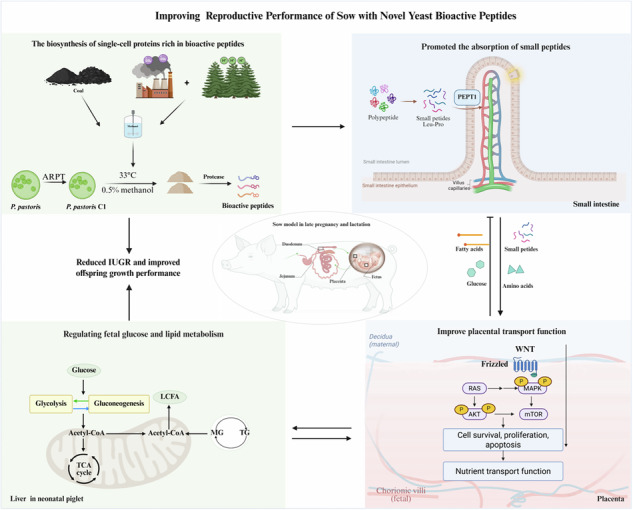

## Introduction

Maternal nutritional status serves as a critical determinant of fetal development and long-term offspring health^1^. Intrauterine growth restriction (IUGR) represents a prominent challenge shared by both livestock production and human nutrition, with its occurrence closely linked to placental dysfunction and inadequate maternal nutritional supply^2,3^. Consequently, the development of functional food ingredients capable of effectively improving maternal nutritional status and optimizing placental transport function holds significant scientific importance and application potential.

Current maternal nutritional interventions are frequently limited by inefficient absorption, low biological targeting accuracy, and insufficient functional integration. In contrast, bioactive peptides (BRs) present a scientifically substantiated alternative, characterized by their low molecular weight, specific absorption mechanisms, and intrinsic dual functionality encompassing both nutritional and regulatory roles^4^, whose health effects depend on their structure and sequence^5,6^. Yeast protein has gained extensive application in the food, feed, and pharmaceutical sectors owing to its balanced nutritional composition, sustainable production, and established safety profile. It has been shown that proteins derived from *Saccharomyces cerevisiae* enhance swine growth performance, immune and placental function, and gut health^7–12^, while also improving lipid metabolism and reducing hepatic fat deposition, thereby boosting overall production efficiency^13,14^. Enzymatic hydrolysis of yeast protein yields peptides with diverse bioactivities, such as antioxidant, immunomodulatory, and glucoregulatory functions, supporting their use in developing functional foods and feeds^6,15,16^. Thus, the application of modern biotechnologies to produce highly active yeast-derived peptides offers a sustainable strategy for developing precision-targeted bioactive ingredients, enhancing their functional value and positioning them as promising candidates for next-generation nutrition solutions.

*Pichia pastoris* is characterized by its high cellular protein content and distinct capacity to utilize non-food, one-carbon substrates, a metabolic trait enabled by its native methanol assimilation pathway. These combined attributes establish it as a uniquely sustainable and productive platform for generating high-quality bioactive peptides^17^. While *P. pastoris* hydrolysates have been explored as alternative foods in crisis scenarios^18^ and as protein supplements in human and animal nutrition^19,20^, with benefits for growth performance and nutrient digestibility in weaned piglets^21^. However, the specific effects and underlying mechanisms of *P. pastoris*-derived peptides on maternal nutrition, placental function, and offspring metabolism remain poorly understood. Thus, this study aims to efficiently produce *P. pastoris*-derived bioactive peptides (PpBP), to elucidate their absorption mechanism through in vitro experiments, evaluate their efficacy in improving reproductive performance in a pregnant sow model, and reveal their underlying mechanisms of action.

## Results

### Creation BPs from *P. pastoris c1* cultured in a bioreactor

**Fig. 1:**
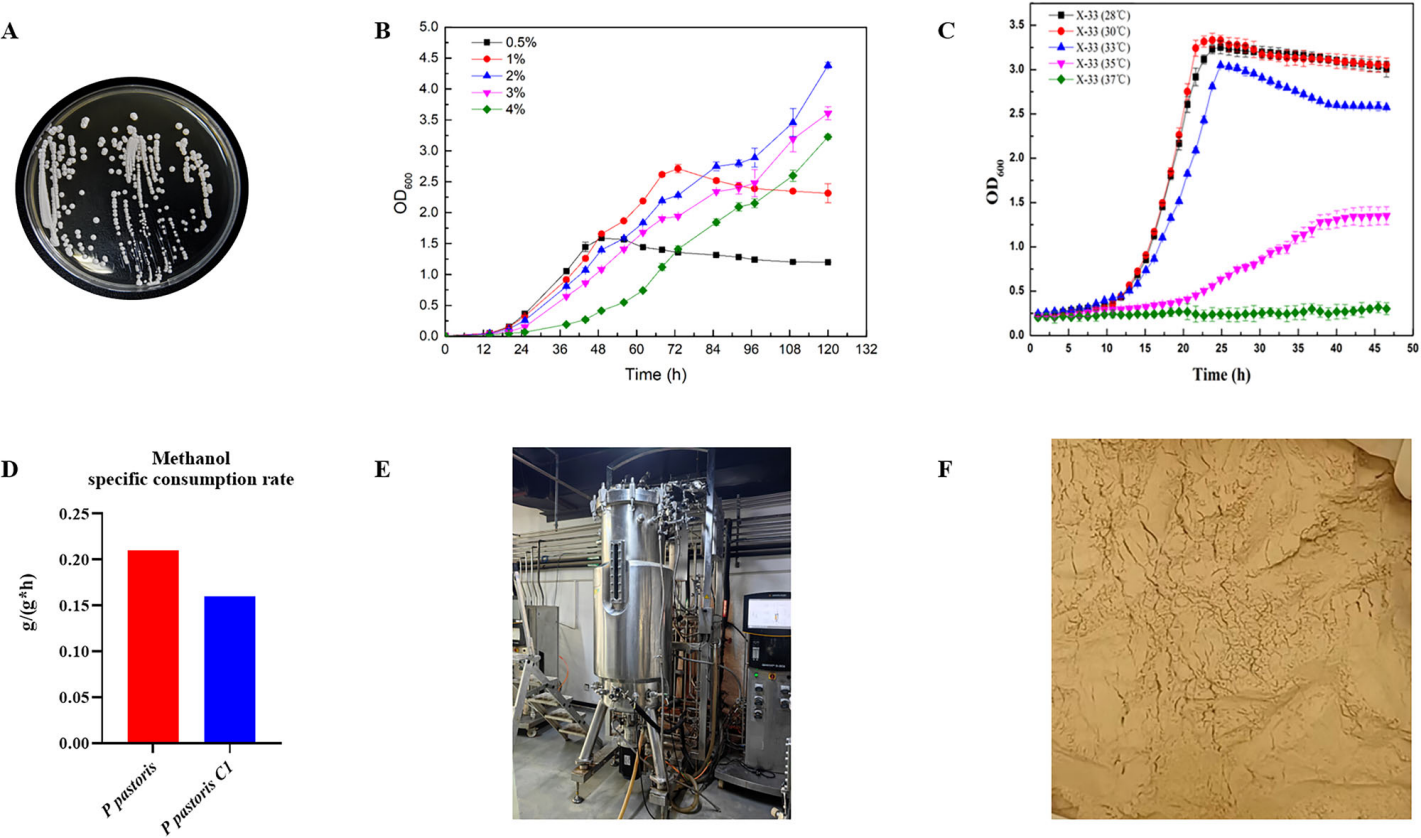
Growth characteristics and process parameters of *P. pastoris.* **A** Representative colony morphology. **B** Growth curve under varying methanol concentrations. **C** Growth curves in 0.5% methanol minimal medium at different temperatures. **D** Specific methanol consumption rate of the mutagenesis-derived strain C1. **E** The pilot-scale bioreactor used in this experiment. **F** enzymatic protein hydrolysate sample after centrifugation, washing and drying.

As shown in Fig. 1B, higher methanol concentrations provided a greater carbon source, leading to a higher maximum biomass. However, they also induced stronger methanol stress, as evidenced by a prolonged lag phase and a reduced slope of the logarithmic growth phase. This growth retardation resulted in a lower specific growth rate. Overall, a methanol concentration of 0.5% (v/v) supported the shortest lag phase and the highest specific growth rate.

The optimal growth temperature for *P. pastoris* was determined to be 28–30 °C. While a suboptimal temperature of 33 °C slightly suppressed growth and 35 °C induced severe inhibition (Fig. 1C). Therefore, 33°C was selected as the preferred temperature for protein production to prioritize product yield over maximal biomass.

After ARTP mutagenesis, we obtained *P. pastoris C1*, a mutant exhibiting the highest methanol utilization rate. Its specific methanol consumption rate reached 0.21 g/(g·h), significantly surpassing the 0.16 g/(g·h) of the parental strain (Fig. 1D).

### Crude protein and peptides profile in PpBP

**Fig. 2:**
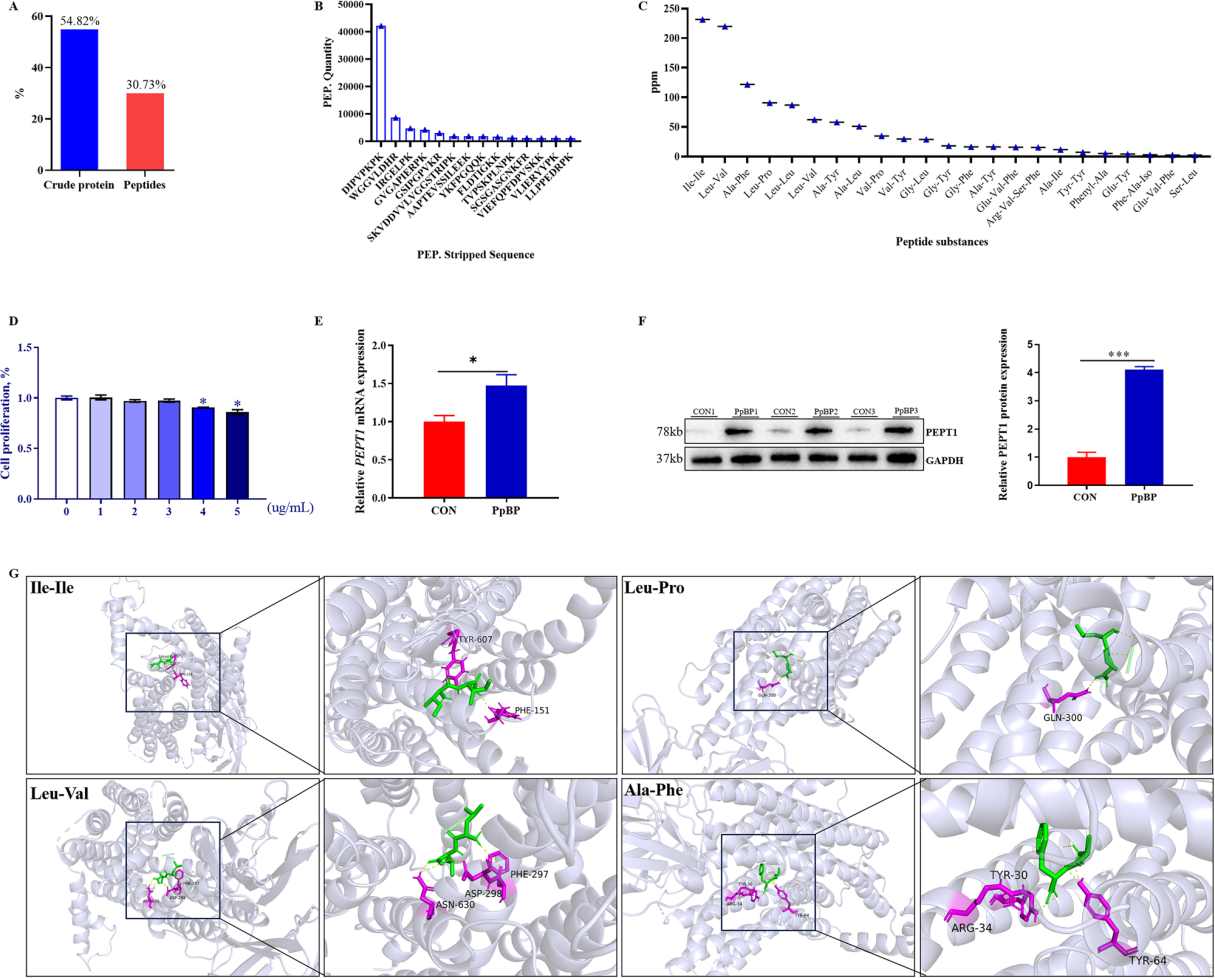
Nutritional composition of *P. pastoris* C1 hydrolyzed peptides and its effect on nutrient transporter expression in IPEC-J2 cells. **A** Crude protein and peptide content. **B** Oligopeptide content. **C** Small peptide content. **D** Cell viability after 24-h PpBP treatment at different concentrations. **E** mRNA expression of PEPT1. **F** Protein expression of PEPT1. **G** Molecular docking between peptides Ile-Ile, Leu-Val, Leu-Pro, Ala-Phe and PEPT1. PepT1 is depicted in cartoon view, and peptides as sticks in the binding pocket. The yellow dashed lines represent the hydrogen bonds. Data are presented as mean ± SEM, *n* = 3–6. Statistical significances were set at *P < 0.05 and ***P < 0.001. *PEPT1* peptide transporter 1.

Quantitative analysis of hydrolyzed protein revealed that crude protein and peptides accounted for 54.82% and 30.73% of the dry cell mass, respectively (Fig. 2A). A total of 165 unique peptide sequences were identified in hydrolyzed protein. Specifically, the oligopeptide fraction was comprised primarily of DIPVPKPK (35.57%), WGGVLDH (7.25%), IRGELPK (4.0%), GVGAPIERPK (3.56%), and GSIPGPVKR (2.54%). Meanwhile, the predominant small peptides were Ile-Ile (231.62 ppm), Leu-Val (219.95 ppm), Ala-Phe (121.77 ppm), Leu-Pro (90.75 ppm), and Leu-Leu (86.92 ppm) (Fig. 2B, C). Notably, these identified peptides collectively exhibit structural features associated with enhanced stability, including hydrophobic C-terminal and a high prevalence of proline residues.

### PpBP regulates intestinal nutrient transporters with functional binding affinity to PEPT1

In comparison to the CON group, PpBP significantly increased the mRNA and protein expression of PEPT1 in the IPEC-J2 (*P* < 0.05, Fig. 2E, F). Molecular docking analysis revealed a clear correlation between Vina scores and the potential for PEPT1 activation, with lower scores indicating stronger binding affinity. The vina scores of Ile-Ile, Leu-Val, Ala-Phe, and Leu-Pro were −6.0, −6.6, −8.4, and −9.0, respectively (Fig. 2G; Table 1). Notably, Leu-Pro, possessing the most favorable score, was found to form key hydrophobic interactions with the active site residue Gln-300. Similarly, Ala-Phe formed bonds with Tyr-30, Arg-34, and Tyr-64, consistent with its strong binding potential.

**Table 1:** Physiochemical characteristics, Vina score, and the binding sites with PEPT1 of peptides

Name	Molecular formula	Molecular weight	Vina score	Hydrogen bond sites interacting with PEPT1
Ile-Ile	C12H24N2O3	244.33 g/mol	−6.0	TYR-607、PHE-151
Leu-Val	C11H22N2O3	230.30 g/mol	−6.6	ASN-630、ASP-298、PHE-297
Ala-Phe	C12H16N2O3	236.27 g/mol	−8.4	TYR-30、ARG-34、TYR-64
Leu-Pro	C_11_H_20_N_2_O_3_	228.29 g/mol	−9.0	GLN-300

### Maternal supplementation with PpBP improved the growth performance of the offspring

The reproductive performance of sows is presented in Tables 2 and 3. In comparison to the CON group, maternal supplementation with PpBP during the late pregnancy led to a significant reduction in IUGR rate (*P* = 0.004). Additionally, when sows were supplemented with PpBP during late pregnancy and lactation, there was a notable increase in the average weight of piglets at weaning as well as the average daily weight gain of the piglets (*P* ≤ 0.05).

**Table 2:** Influence of maternal PpBP supplementation on reproductive performance of sows

Items	CON group			PpBP group			*P* value		
	*n*	Avg ± SD	CV (%)	*n*	Avg ± SD	CV (%)	Diet	BF	Diet *BF
Litter size, *n*	20	13.85 ± 2.581	18.63	20	14.90 ± 2.918	19.59	0.317	0.361	0.418
Number born alive, *n*	20	12.95 ± 2.235	17.26	20	13.75 ± 2.531	18.41	0.463	0.639	0.738
Birth mortality (%)^a^	20	6.59 ± 4.409	66.88	20	7.07 ± 4.881	69.04	0.304	0.486	0.135
IUGR rate (%)^b^	20	15.50 ± 5.797	37.40	20	5.62 ± 5.182	92.12	0.004	0.612	0.635
Birth litter weight, kg	20	15.78 ± 2.921	18.51	20	17.57 ± 3.215	18.30	0.850	0.735	0.901
Birth weight, kg	20	1.23 ± 0.223	18.09	20	1.28 ± 0.139	10.8172592	0.221	0.248	0.750

^a^Birth mortality = (total litter size–alive)/total × 100^b^. IUGR rate = the number of IUGR/total × 100. IUGR piglets were defined as those with a birth weight ≤65% of the heaviest littermate’s weight within the same litter. Data are presented as mean ± standard deviation (SD), with the group coefficient of variation (CV, %) shown in parentheses.

**Table 3:** Impact of maternal PpBP supplementation on growth performance of piglets

Items	CON group			PpBP group			*P* value
	*n*	Avg ± SD	CV (%)	*n*	Avg ± SD	CV (%)	
Initial litter size, *n*	15	13.50 ± 1.743	12.91	15	14.42 ± 2.738	18.97	0.294
Initial litter weight, kg	15	16.79 ± 4.326	25.77	15	18.23 ± 3.421	18.76	0.335
Initial weight, kg	15	1.22 ± 0.229	18.73	15	1.27 ± 0.101	7.92	0.403
Litter size at weaning, *n*	15	9.73 ± 2.520	25.89	15	10.20 ± 2.042	20.02	0.582
Litter weight at weaning, kg	15	55.46 ± 16.436	29.64	15	64.18 ± 14.878	23.18	0.139
Average weight of piglet at weaning, kg	15	5.70 ± 0.661	11.60	15	6.28 ± 0.845	13.46	0.043
Average daily weight gain, kg	15	0.224 ± 0.030	13.36	15	0.250 ± 0.0404	16.15	0.050

Data are presented as mean ± SD, with the group coefficient of variation (CV, %) shown in parentheses.

### PpBP supplementation altered the serum metabolic profile of sows by predominantly affecting lipid metabolism

**Fig. 3:**
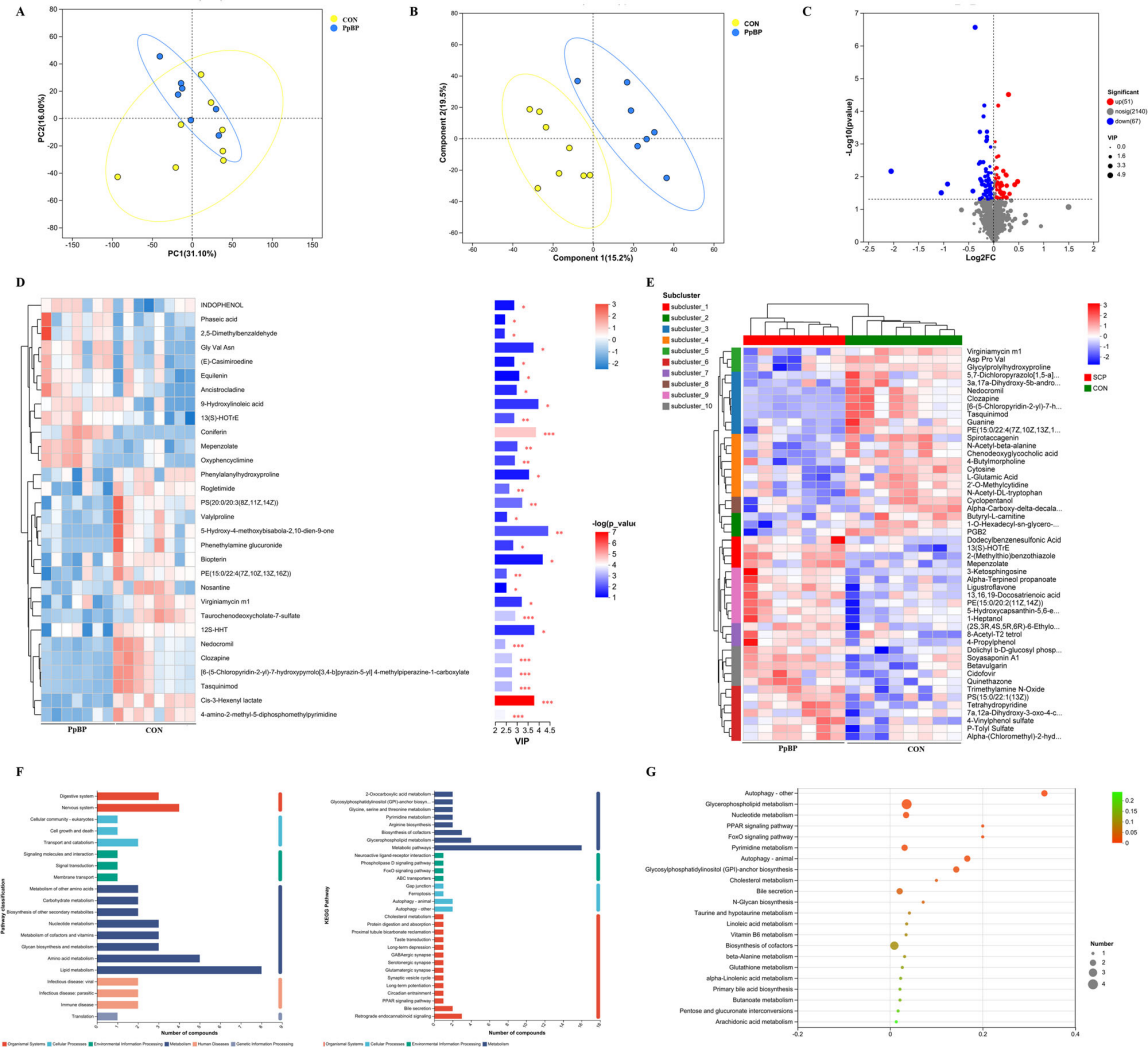
Effects of dietary PpBP supplementation on serum metabolomic profiles in sows. **A** Principal component analysis (PCA) score plot. **B** Partial least squares-discriminant analysis (PLS-DA) score plot. **C** Volcano plot of differential metabolites. **D** Variable importance in projection (VIP) score bar plot. **E** Heatmap of hierarchical clustering analysis for metabolite profiles. **F** KEGG pathway analysis of differential metabolites, **G** KEGG pathway enrichment between PpBP and CON groups.

Untargeted metabolomics analysis revealed significant serum metabolite variations in sows. OPLS-DA and PCA showed clear group separations (Fig. 3A, B), with a volcano plot highlighting differential metabolites (Fig. 3C). Based on VIP > 1 and *P* < 0.05, PpBP treatment elevated 51 metabolites and reduced 67 compared to CON. Notably, PpBP treatment significantly altered serum metabolite levels, elevating key compounds like Gly-Val-Asp and 13,16,19-Docosatrienoic acid while reducing others, including Asp-Pro-Val, Gly-Leu, Asp-Leu, N-Acetyl-L-methionine, N-Acetyl-DL-tryptophan, N-Acetyl-L-glutamic acid, N-Acetyl-beta-alanine, and L-glutamic acid (*P* < 0.05; Fig. 3D, E). These differential metabolites were primarily associated with amino acid, lipid and carbohydrate metabolism (Fig. 3F). More specifically, the primary biological functions of the serum differential metabolites between the CON and PpBP groups encompassed glycerophospholipid metabolism, cholesterol metabolism, bile secretion, pyrimidine metabolism, D-amino acid metabolism, glycosylphosphatidylinositol (GPI)-anchor biosynthesis, the PPAR and FoxO signaling pathway (*P* < 0.05, Fig. 3G).

### PpBP supplementation improved maternal and umbilical cord blood lipid profiles

Compared to the CON group, maternal PpBP supplementation significantly reduced serum TG level in sows (*P* < 0.05), increased umbilical cord serum concentrations of TC, LDL-C3, and HDL-C4 (*P* < 0.05). Moreover, maternal PpBP supplementation showed a tendency to decrease serum total TBA in sows (*P* = 0.077, Table 4).

**Table 4:** Effects of dietary PpBP supplementation on serum biochemical parameters in sows, umbilical cord blood, and neonatal piglets

Items	The serum of a pregnant sow		*P* value	The serum of the umbilical cord		*P* value	The serum of piglets		*P* value
	CON	PpBP		CON	PpBP		CON	PpBP	
GLU, mmol/L	4.44 ± 0.633	4.65 ± 0.589	0.813	2.20 ± 0.400	1.62 ± 0.305	0.276	5.50 ± 0.348	4.96 ± 0.471	0.378
TG, mmol/L	0.59 ± 0.077	0.40 ± 0.027	0.043	0.32 ± 0.058	0.28 ± 0.058	0.679	0.54 ± 0.094	0.49 ± 0.084	0.687
TC, mmol/L	2.23 ± 0.151	1.95 ± 0.150	0.218	0.94 ± 0.030	1.30 ± 0.151	0.042	1.60 ± 0.230	1.41 ± 0.145	0.504
LDL-C3, mmol/L	1.07 ± 0.074	0.95 ± 0.069	0.289	0.43 ± 0.036	0.65 ± 0.085	0.038	0.74 ± 0.052	0.51 ± 0.036	0.790
HDL-C4, mmol/L	0.90 ± 0.126	0.89 ± 0.067	0.944	0.26 ± 0.033	0.43 ± 0.061	0.041	0.52 ± 0.196	0.54 ± 0.045	0.918
NEFA, mmol/L	0.37 ± 0.136	0.39 ± 0.149	0.961	0.09 ± 0.025	0.09 ± 0.018	0.831	0.27 ± 0.062	0.20 ± 0.050	0.405
TBA, μmol/L	24.98 ± 5.539	13.18 ± 2.250	0.077	24.92 ± 6.967	25.25 ± 1.950	0.965	19.07 ± 4.120	17.78 ± 4.793	0.844

Data are presented as mean ± SEM, *n* = 8. *GLU* glucose, *TG* total triglyceride, *CHOL* cholesterol, *LDL* low-density lipoprotein; HDL high-density lipoprotein, *NEFA* nonestesterified fatty acid, *TBA* total bile acid.

### PpBP modulated placental gene networks, signaling pathways, and nutrient transport

**Fig. 4:**
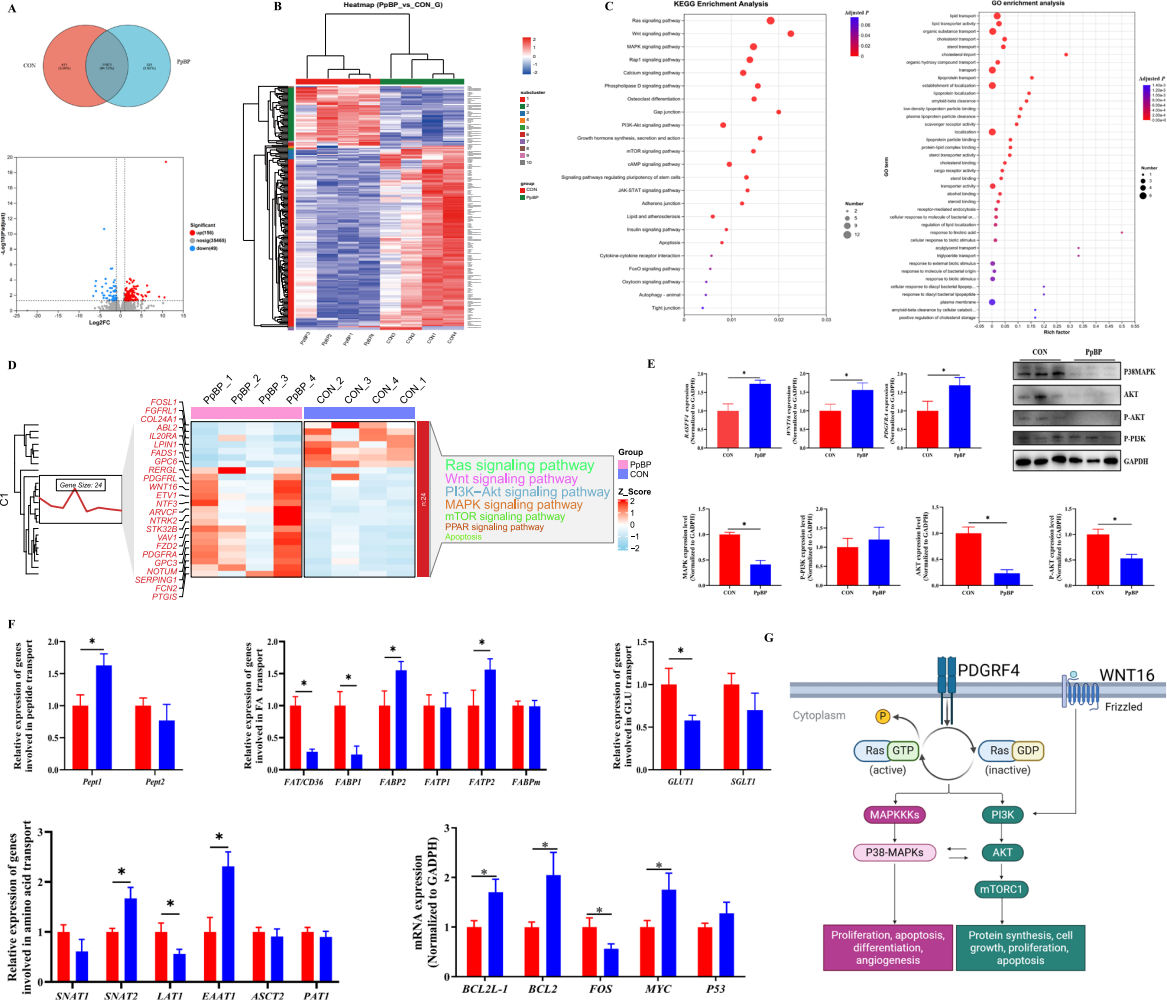
Effects of dietary PpBP supplementation on placental function in sows. **A** Venn diagram and volcano plot of differentially expressed genes (DEGs). **B** PpBP-induced DEGs in the placenta. **C** KEGG and GO enrichment analysis of DEGs. **D** PpBP-induced DEGs associated with key placental signaling pathways. **E** mRNA and protein expression of genes related to RAS, WNT, MAPK, and AKT signaling pathways. **F** mRNA expression of genes involved in placental transport, apoptosis, and proliferation. **G** Schematic illustration of the mechanism of PpBP on the placental pathway (Created in BioRender.com). Data are presented as mean ± SEM (*n* = 3–8). Statistical significance was set at *P < 0.05. RASSF4 retinoic acid-secreted frizzled-related protein 4, WNT16 wnt family member 16, PDGFRA platelet-derived growth factor receptor alpha, GLUT1 glucose transporter 1, SGLT-1 Na + -dependent glucose transporter 1, FAT/CD36 fatty acid transporter/CD36, FABP1 fatty acid-binding protein 1, FABP2 fatty acid-binding protein 2, FATP1 fatty acid transport protein 1, FATP2 fatty acid transport protein 2, FABPpm fatty acid-binding protein, PEPT1 peptide transporter 1, PEPT2 peptide transporter 2, SNAT2 sodium-dependent neutral amino acid transporter 2, LAT1 Large Neutral Amino Acid Transporter 1, EAAT1 excitatory amino acid transporter 1, ASCT2 alanine-serine-cysteine transporter 2, PAT1 proton-coupled amino acid transporter 1. BCL2l-1 B-cell lymphoma 2-like 1, BCL2 B-cell lymphoma 2, FOS Fos proto-oncogene AP-1 transcription factor subunit, MYC MYC proto-oncogene, P53 tumor protein P53.

To investigate the regulatory impact of PpBP on placental gene networks and key metabolic pathways, we performed transcriptome sequencing. PCA revealed distinct clustering between the PpBP and CON groups, indicating significant transcriptional differences. Differential expression analysis identified 156 upregulated and 49 downregulated genes in the PpBP group (Fig. 4A, B). KEGG enrichment analysis highlighted significant involvement of these genes in critical signaling pathways, including apoptosis, autophagy, and Ras, Wnt, MAPK, Rap1, PI3K-Akt, mTOR, cAMP, and VEGF signaling pathway (*P* < 0.05). Additionally, GO analysis demonstrated strong associations with lipid metabolism, particularly in transport processes (cholesterol, sterol, triglyceride, phospholipid, lipoprotein) and cellular uptake (long-chain FA (LCFA) import, intestinal cholesterol/lipid absorption) (*P* < 0.05, Fig. 4C, D).

Consistent with transcriptome results, PpBP significantly upregulated placental mRNA levels of retinoic acid-secreted frizzled-related protein 4 (RASSF4), wnt family member 16 (WNT16), platelet-derived growth factor receptor alpha (PDGFRA), while downregulating P38 MAPK, AKT, and p-AKT protein expression (*P* < 0.05, Fig. 4E). It also increased placental mRNA expression of *Pept1*, *FABP2*, *FATP2*, *SNAT1*, *EAAT1*, while downregulating *GLUT1*, *FAT/CD36, FABP1* and *LAT1* (*P* < 0.05). Additionally, PPBP altered genes regulating proliferation and apoptosis, such as B-cell lymphoma 2-like 1(BCL2l-1), B-cell lymphoma 2 (BCL2), Fos proto-oncogene, AP-1 transcription factor subunit (FOS), MYC proto-oncogene (MYC) (*P* < 0.05; Fig. 4F).

### Maternal PpBP supplementation enhanced hepatic gluconeogenesis in neonatal piglets

**Fig. 5:**
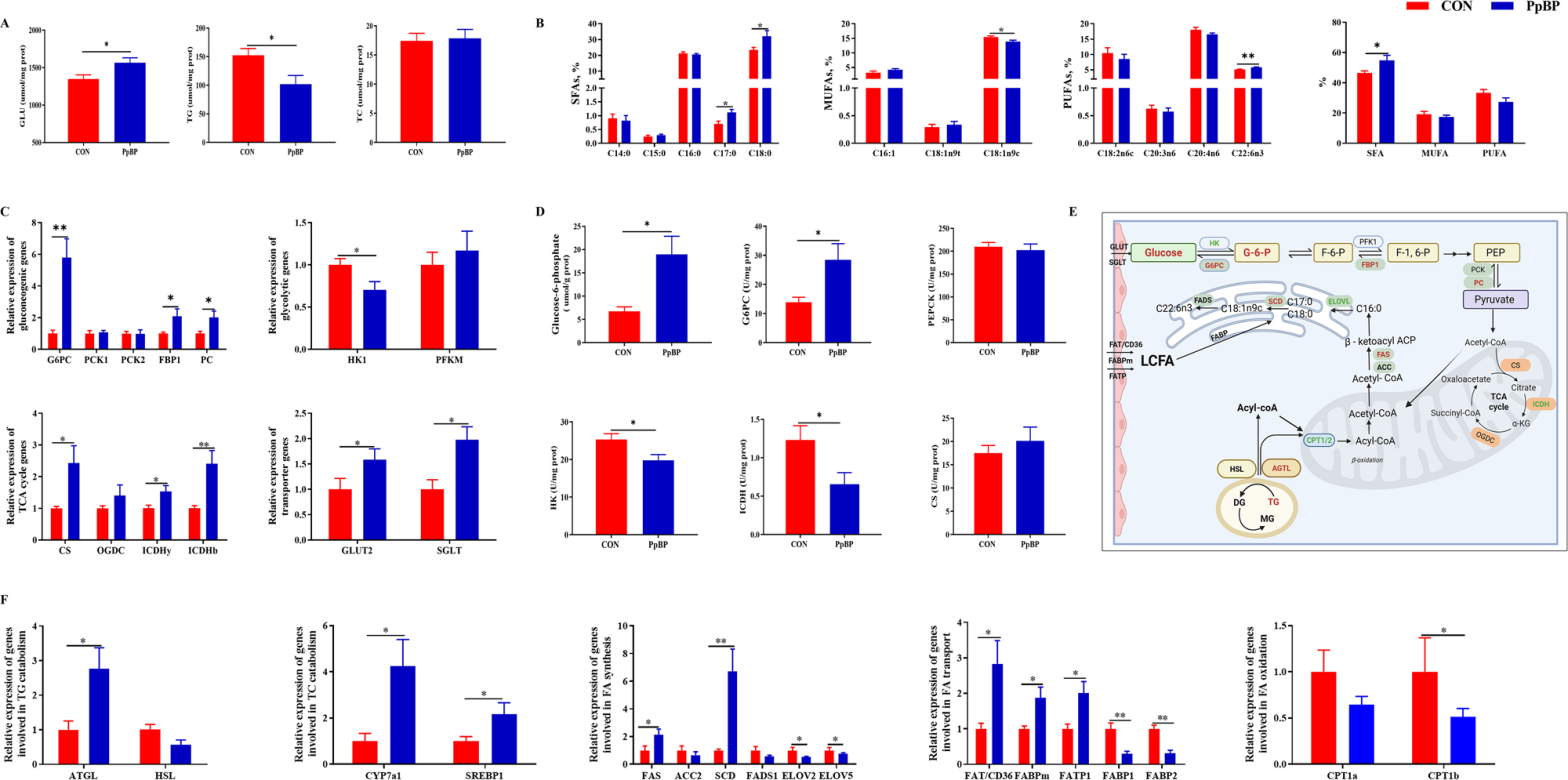
Effects of maternal PpBP supplementation on hepatic glucose and triglyceride metabolism in neonatal piglets. **A** Hepatic GLU, TG, and TC concentrations in neonatal piglets. **B** Hepatic FA composition in neonatal piglets. **C** Hepatic mRNA expression of rate-limiting enzymes in glucose metabolism. **D** Hepatic enzymatic activity of rate-limiting enzymes in glucose metabolism. **E** Schematic illustration of the mechanism of PpBP on glucose and lipid metabolism (Created in BioRender.com). **F** hepatic mRNA expression of genes involved in TC and TG metabolism. GLU glucose, TG triglyceride, TC total cholesterol, SFAs saturated fatty acids, MUFAs monounsaturated fatty acids, PUFAs polyunsaturated fatty acids, G6PC glucose-6-phosphatase, PCK1 phosphoenolpyruvate carboxykinase 1, PCK2 phosphoenolpyruvate carboxykinase 2, PC pyruvate carboxylase, FBP1 fructose 16-bisphosphatase 1, HK1 hexokinase, pFkm phosphofructokinase, CS citrate synthase, OGDC ketoglutarate dehydrogenase complex, ICDH-β socitrate dehydrogenase β, ICDH-γ socitrate dehydrogenase γ, GLUT2 glucose transporter 2, SGLT-1 Na + -dependent glucose transporter 1, PEPCK phosphoenolpyruvate carboxykinase, CS citrate synthase, ICDH isocitrate dehydrogenase, FAT/CD36 fatty acid transporter/CD36, FABP1 fatty acid binding protein 1, FABP2 fatty acid binding protein 2, FATP1 fatty acid transport protein 1, FATP2 fatty acid transport protein 2, FABPpm fatty acid-binding protein, ATGL adipose triglyceride lipase, HSL hormone-sensitive lipase, CPT1-α carnitine palmitoyl transferase1-α, CPT1-β carnitine palmitoyl transferase1-β, FAS fatty acid synthase, ACC2 acetyl-CoA carboxylase acetyl-CoA carboxylase 2, SCD stearoyl coenzyme A desaturase, ELOVL2 fatty acid elongase 2, ELOVL5 fatty acid elongase 5, FADS1 fatty acid desaturase 1, Cyp7α1 cholesterol- 7a-hydroxylase, SREBP1c sterol regulatory element-binding protein 1.

Compare to the CON group, maternal supplementation with PPBP significantly increased GLU and G6P levels in the liver of neonatal piglets (*P* < 0.05, Fig. 5A, D). Maternal PpBP supplementation induced significant alterations in hepatic gene expression and enzyme activity in neonatal piglets (Fig. 5C). Specifically, it significantly upregulated gluconeogenic genes such as G6PC, fructose 1,6-bisphosphatase 1 (FBP1), and pyruvate carboxylase (PC), as well as genes involved in the TCA cycle including citrate synthase (CS), isocitrate dehydrogenase (ICDH) β, ICDH-γ, and glucose transporters such as glucose transporter 2 (GLUT2) and Na⁺-dependent glucose transporter 1 (SGLT-1), while downregulating HK1 (*P* < 0.05). Enzymatically, maternal PPBP supplementation significantly enhanced G6PC activity and reduced ICDHm and HK activity in the livers of neonatal piglets (*P* < 0.05, Fig. 5D).

### Maternal PpBP shifts hepatic lipid metabolism from oxidation toward synthesis in neonatal piglets

In comparison to the CON group, maternal supplementation with PPBP significantly decreased TG level in the liver of neonatal piglets (*P* < 0.05, Fig. 5A). Additionally, maternal supplementation with PpBP significantly elevated concentrations of C17:0, C18:0, C22:6n3 fatty acids (FAs) profile, and reduced C18:1n9c level in the liver of neonatal piglets (*P* < 0.05, Fig. 5B).

Maternal PpBP supplementation induced significant alterations in hepatic gene expression and enzyme activity related lipid metabolism in neonatal piglets (Fig. 5F). Specifically, PpBP enhanced lipogenic genes such as adipose triglyceride lipase (ATGL), FA synthase (FAS), stearoyl coenzyme A desaturase (SCD), as well as genes involved in the cholesterol metabolism including cholesterol- 7a-hydroxylase (Cyp7α1), sterol regulatory element-binding protein 1 (SREBP1), and FA transporters including FA transporter/CD36 (FAT/CD36), FA transport protein 1(FATP1), FA-binding protein (FABPpm). Conversely, PpBP suppressed FA oxidation genes such as carnitine palmitoyl transferase1-β (CTP1β), FA elongase (ELOV) 2, ELOV5, and FA transporters, including FA-binding protein 1 (FABP)1 and FABP2 in the livers of neonatal piglets (*P* < 0.05).

## Discussion

Yeast is a primary and valuable source of BPs, yet realizing their full potential requires the establishment of efficient and scalable production systems^5^. The methylotrophic yeast *P. pastoris* offers a distinct biotechnological advantage in this regard, as its ability to efficiently utilize methanol as a sole carbon and energy source allows the conversion of low-cost industrial by-products into high-value BPs. In this study, we developed an optimized production platform for *P. pastoris*-derived BPs. Fermentation kinetics analysis revealed a double-edged sword effect of methanol, and a concentration of 0.5% (v/v) was identified as optimal for balancing rapid adaptation and efficient growth by minimizing methanol-induced stress. Further temperature optimization highlighted a critical trade-off between growth and production, leading to the adoption of a suboptimal temperature (33 °C) that favored product synthesis over maximal biomass. To enhance process efficiency, we generated a mutant strain, C1, via ARTP mutagenesis, which exhibited a 31% increase in specific methanol consumption rate. Crucially, our comprehensive characterization of the PpBP bridged its production with its biological function. Compositional analysis demonstrated high conversion efficiency, with crude protein and oligopeptides accounting for 54.82% and 30.73% of the dry cell weight, respectively. Peptide profiling by LC-MS/MS further identified a rich array of specific oligopeptides (e.g., DIPVPKPK) and dipeptides, including Ile-Ile, Ala-Phe, and notably, Leu-Pro.

The small intestine serves as the primary site for peptide absorption, with PEPT1 (a member of the solute carrier family 15, SLC15) mediating the uptake, distribution, and resorption of di- and tripeptides throughout the body^22^. Our findings that PpBP significantly upregulates PEPT1 expression indicated that yeast-derived small peptides enhance their own intestinal absorption^23^. This observation underscores the role of yeast-derived small peptides in promoting their bioavailability via facilitated uptake in the small intestine. Molecular docking was employed to predict possible interactions between representative dipeptides (e.g., Leu-Pro, Ala-Phe) and the PEPT1 binding pocket. A lower Vina score indicates stronger predicted binding affinity, which may correlate with a higher likelihood of transporter activation^24^. The predicted binding poses revealed that Leu-Pro formed hydrophobic contacts and a hydrogen bond with Gln-300, while Ala-Phe established three hydrogen bonds with Tyr-64, Tyr-30 and Arg-34. These in silico results suggest that strong interaction with key residues (e.g., Phe-297, Val-626, Trp-622, Tyr-64, Asn-329, Gln-300) within the active site could promote PEPT1 function^23^. It should be noted, however, that these docking predictions require further experimental validation to confirm whether the predicted binding translates into physiologically relevant transport activity. Recent approvals of yeast hydrolysates from various species as dietary supplements underscore their value as safe, high-quality functional protein sources^25,26^. In swine, yeast and its derivatives have been shown to enhance growth performance and immune function^27,28^. Notably, *S. cerevisiae* hydrolysate has proven particularly effective, not only improving reproductive performance and litter weight by modulating nutrient metabolism^12^, but also enhancing growth and digestibility in both piglets and growing pigs^29–31^. The high abundance of small peptides in these yeasts is well-established. Therefore, it is plausible that these peptides contribute significantly to the observed regulatory effects on animal healthy.

In the present study, the improved growth performance of offspring, manifested as reduced IUGR incidence and increased weaning weight and daily gain. Given that PpBP was administered via the maternal diet, it is plausible that its beneficial effects are mediated through maternal and/or placental adaptations. Firstly, absorbed peptides may modulate systemic metabolism to enhance the nutrient supply available for placental transfer. Alternatively, bioactive components (or metabolites) may directly influence placental development, transporter expression, or signaling pathways to increase nutrient delivery efficiency to the fetus. The abundance of peptides in PpBP featuring hydrophobic C-termini and proline-rich sequences is likely to enhance their structural stability and bioavailability, which could facilitate either systemic maternal effects or placental interaction^32^. Therefore, these peptides could modulate the activity of key metabolic enzymes in the mother or placenta, ultimately influencing nutrient metabolism and allocation to the fetus. However, future studies need to determine if these small peptides in PpBP can cross the placenta to act directly on fetal tissues. In summary, the benefits of PpBP supplementation are consistent with its unique peptide profile, likely mediated through maternal or placental adaptations.

Biochemical indices and serum metabolites serve as reliable indicators of metabolic status in late-pregnancy sows. In the present study, dietary supplementation with PpBP significantly elevated serum levels of Gly-Val-Asp while reducing concentrations of multiple small peptides and acetylated amino acids, including Val-Pro, Gly-Leu, Asp-Leu, and N-acetylated derivatives of tryptophan, glutamic acid, methionine, and beta-alanine. This metabolic profile indicates the altered metabolic dynamics of small-molecule nitrogen sources in late-pregnancy sows, which could involve changes in absorption, turnover, or utilization efficiency. The observed shifts suggest that PpBP supplementation was associated with regulated nitrogen metabolic status. This may be attributed to the provision of highly stable, bioavailable peptides that improved dietary nitrogen quality and the potential generation of specific bioactive metabolites, such as Gly-Val-Asp, which could act as direct modulators of metabolic pathways. Furthermore, supplementation with PpBP induced a broad rewiring of lipid metabolism, impacting key pathways such as glycerophospholipid metabolism, GPI-anchor biosynthesis, and cholesterol metabolism. This metabolic shift was evidenced by specific alterations in lipid species, including elevated levels of PE (15:0/20:2(11Z,14Z)) and PS (15:0/22:1(13Z)), alongside reductions in chenodeoxyglycocholic acid, PE (15:0/22:4(7Z,10Z,13Z,16Z)), and PS (20:0/20:3(8Z,11Z,14Z)). Consistently, serum biochemical analysis confirmed a significant decrease in maternal TG levels. Given that PpBP itself contributes negligible lipids, these lipid-lowering effects are likely mediated by other bioactive constituents, with its rich peptide content being a prime candidate for regulating metabolic homeostasis. Since elevated maternal triglycerides are associated with adverse pregnancy outcomes, including excessive fetal growth^33^, our findings suggested that PpBP supplementation may confer benefits by ameliorating lipid metabolism. In conclusion, our study indicated that the beneficial effects of PpBP are primarily associated with the modulation of small peptide and lipid metabolic pathways.

The umbilical cord serves as a vital connection between the fetus and the mother, supplying essential oxygen and nutrients to the developing fetus. In this study, maternal PpBP supplementation elevated umbilical cord serum levels of TC, LDL-C, and HDL-C, indicating modulated cholesterol metabolism. Cholesterol, a critical component of cell membranes, serves as a precursor for steroid hormones, vitamin D, and bile acids, is distributed to tissues via LDL and recycled to the liver via HDL^34^. The observed increases in these lipids likely reflect heightened fetal demand for growth. Furthermore, PpBP supplementation also reduced hepatic TG while increasing GLU levels in neonatal piglets, suggesting a modulation of energy metabolism. Metabolic switch from glycogen to lipid in the liver maintains GLU homeostasis in neonatal mice at day 1 of lactation for 3–4 h^35^. Thus, the observed metabolic changes are consistent with a pattern in which PpBP supplementation is associated with the conversion of TG to GLU, which may contribute to increased GLU availability. Since FAs constitute the fundamental components of TGs, we further observed that maternal PpBP supplementation elevated the proportions of C17:0 and C18:0 FAs in the liver of neonatal piglets, suggesting an increased ratio of SFAs. Notably, PpBP supplementation also enhanced C22:6n3 (docosahexaenoic acid, DHA) while reducing C18:1n9c levels in the hepatic tissue. DHA, being an essential FA, plays a critical role in fetal neurodevelopment and brain maturation. Together, these results demonstrated that maternal PpBP supplementation during late pregnancy regulated TC, TG, and GLU metabolism in the feto-placental unit.

The placenta serves as the critical interface between maternal and fetal circulatory systems, facilitating essential nutrient/waste exchange to support fetal development^36^. Our findings suggest that maternal PpBP supplementation modulated the expression of genes associated with placental development and function. Specifically, FOSL1, NTF3, and PDGFRA, which are involved in cellular proliferation and differentiation, were upregulated in the PpBP group. Additionally, PpBP supplementation regulated *ABL2* and *NTRK2* expression, which are involved in critical signaling pathways (PI3K-AKT, RAS-MAPK) that control cellular proliferation, survival, and differentiation. Together, these transcriptional changes indicated that PpBP may influence molecular pathways relevant to placental growth and function. Transcriptomic analysis revealed that PpBP-modulated genes were enriched in pathways including Ras, Wnt, MAPK, PI3K-Akt, mTOR signaling. Specifically, PpBP supplementation upregulated multiple Wnt pathway components (e.g., *WNT16*, *FZD2*), suggesting that the Wnt signaling pathway may be altered under these experimental conditions. Furthermore, PpBP supplementation increased expression of *RASSF4* (a Ras activator), *PDGFRA* and *NTRK2*, suggested PpBP is involved in altering both the Ras/MAPK and PI3K/AKT signaling pathways. Interestingly, phosphorylation levels of p38 MAPK and AKT were decreased following PpBP supplementation, a finding that may point to complex regulatory interactions within the signaling network. These findings suggested a link between maternal PpBP supplementation and alterations in placental development. These pathways could be involved in regulating trophoblast proliferation, differentiation, and apoptosis, although this interpretation requires further validation. Consistently, PpBP supplementation increased the gene expression of *BCL2l-1*, *BCL2*, *MYC*, and decreased the gene expression of *FOS*, and these transcriptional changes are associated with the regulation of apoptosis, proliferation and differentiation. Placental proliferation and differentiation are intrinsically linked to its functional capacity^37^. The present study demonstrated that PpBP supplementation significantly modulated the expression of genes associated with small peptides, lipid and glucose transport in the placenta. In the present study, PpBP increased the gene expression of *MSR1*, *PLSCR4*, *ABCA6*, *OSBPL6*, and decreased the gene expression of *STARD5*, which are primarily involved in lipid, cholesterol, and sterol transport pathways, suggesting that PpBP supplementation may influence the expression of genes related to placental lipid transport. Moreover, PpBP treatment increased the expression of *FABP2*, *FATP2*, but decreased the expression of *FAT/CD36*, *FABP1* and *GLUT1*. These coordinated changes indicated PpBP could regulate the placental GLU and FA transfer at the transcriptional level.

The liver serves as a central regulator of GLU and lipid metabolism. Lipid metabolism involves a series of transport carriers and rate-limiting enzymes. In the present study, PpBP supplementation was associated with elevated *ATGL* expression, a change consistent with the changed TG hydrolysis. Additionally, the upregulation of *FAS* and *SCD* expression, the genes encoding key enzymes in SFA and MUFA fatty acid synthesis, suggests that PpBP may influence fatty acid synthetic pathways. Consistent with this, SREBP1c, which activates genes like *FAS* and *SCD*^38^, may contribute to the increased hepatic SFA levels due to its higher expression. ELOVL3 and ELOVL5, enzymes involved in DHA synthesis, were downregulated. This reduced expression coincides with elevated hepatic DHA levels in neonatal piglets and may correspond to a homeostatic or feedback response. For FA transport, FAT/CD36 and FATP1 facilitate LCFA uptake, while FABP1/2 shuttle LCFAs to mitochondria, and FABPm further mediates intracellular LCFA transport, delivering FAs to the mitochondrial matrix for β-oxidation. In this study, maternal PpBP supplementation upregulated *FAT/CD36*, *FATP1*, and *FABPm* but downregulated *FABP1/2*, indicating altered FA transport. Furthermore, the lower expression of *CPT1β*, a gene essential for facilitating LCFA entry into mitochondria for β-oxidation, is consistent with a potential downregulation of FA oxidative pathways at the transcriptional level following PpBP supplementation. Collectively, the observed changes in gene expression following PpBP supplementation suggested that it may modulate hepatic lipid metabolism.

Hepatic GLU and TG metabolism are intrinsically interconnected through shared metabolic intermediates, coordinated hormonal regulation, and integrated energy-sensing mechanisms. GLUT2 and SGLT mediate GLU absorption and transport. Their upregulated expression is consistent with a potential increase in hepatic glucose uptake and transport at the transcriptional level following PpBP supplementation. The liver primarily metabolizes GLU through three key pathways: gluconeogenesis, glycolysis, and the TCA cycle. Notably, we observed that PpBP supplementation upregulated the expression of two critical gluconeogenic enzymes - FBP1, which catalyzes the conversion of fructose-1,6-bisphosphate to fructose-6-phosphate, and G6PC, responsible for the final step of GLU production from glucose-6-phosphate. Collectively, these gene and enzyme expression changes suggested that PpBP supplementation may influence hepatic glucose metabolism, particularly through modulation of gluconeogenic networks. Conversely, PpBP supplementation downregulated *HK1* expression, the enzyme that initiates glycolysis. Together with the upregulation of gluconeogenic genes *FBP1* and *G6PC*, these transcriptional changes suggest a coordinated shift in hepatic glucose metabolism that may regulate glucose availability, even in the context of an observed rise in glucose-6-phosphate levels. These coordinated shifts may increase the availability of substrates for hepatic glucose metabolism, suggesting a potential metabolic adaptation that could support fetal development^39^. The scope of this study was focused on the role of PpBP in maternal reproductive and offspring outcomes. Future work to validate additional functionalities of these yeast-derived short-chain peptides, such as their antioxidant and immunomodulatory activities, will further elucidate their functional profile. Additionally, while comparative analysis of peptides from different microbial strains remains a pertinent future direction, this study provides a foundational model by establishing a complete workflow from strain development to in vivo mechanistic validation.

## Methods

### Isolation and identification of *P. pastoris*

A yeast strain was isolated from winery-derived grape pomace. The sample was suspended in sterile water with glass beads and shaken at 30°C for 30 min. The resulting suspension was serially diluted (10^−1^ to 10^−7^), and aliquots from the 10^−5^ to 10^−7^ dilutions were spread onto agar plates with methanol as the sole carbon source. After incubation at 30 °C for 5 days, a pure isolate was obtained through repeated streaking and was identified as *P. pastoris* based on morphological and molecular characteristics.

### Automated analysis of microbial growth curves

The growth of *P. pastoris* was monitored using a fully automated microbial growth curve analyzer. Briefly, a single colony was inoculated into 3 mL of YPD medium and incubated overnight at 30-33 °C with shaking at 220 rpm. The resulting culture was then transferred into 100 mL of Delft medium supplemented with 0.5% methanol in a 250 mL flask and grown to mid-log phase (OD₆₀₀ = 0.5–0.8). Cells were aseptically harvested by centrifugation at 500 × g for 5 min, resuspended in fresh Delft medium, and the OD₆₀₀ was measured. The cell suspension was subsequently inoculated into a 48-well plate containing 1 mL of Delft medium with different methanol level per well, with an initial OD₆₀₀ adjusted to 0.25. The plate was then placed in the automated growth analyzer, and cell growth was recorded under continuous shaking at 800 rpm and 30-33 °C to generate growth curves. Each strain was assayed in six replicates.

### ARTP mutagenesis and high-throughput screening of *P. pastoris*

*P. pastoris* cells cultured in methanol for 3 days were harvested, washed with PBS, and resuspended to approximately 10⁷ CFU·mL⁻¹. A 10 μL aliquot of the suspension was spread onto a sterile metal slide and subjected to ARTP mutagenesis at an input power of 120 W and a gas flow rate of 10 SLM, with exposure times ranging from 80 to 200 seconds. Following treatment, the cells were recovered in 1 mL of PBS, allowed to stand for 2–3 hours, and then plated on YPD agar after appropriate dilution. Surviving colonies were picked into 96-deep-well plates containing methanol-based MM medium. After 30 hours of cultivation, residual methanol concentration was measured, and the mutant strain C1, which exhibited the highest methanol consumption rate, was selected for further study. The fungal strain was preserved in China General Microbiological Culture Collection Center (CGMCC NO. 24324). Unless otherwise specified, *P. pastoris C1* were cultivated in YPD medium containing 20 g/L glucose, 20 g/L peptone and 10 g/L yeast extract.

### Pilot-scale production of *P. pastoris C1* for hydrolyzed peptides synthesis

*P. pastoris* C1 were initially cultivated in YPD or methanol-based Delft medium, followed by adaptive laboratory evolution in sealed flasks through approximately 100 serial subcultures to enhance methanol tolerance and cell density. The evolved strain was subsequently scaled up using a 500 L automatic fed-batch bioreactor (Shanghai Bailun Biotechnology Co., Ltd)^40,41^. The primary batch fermentation was conducted in a Delft medium supplemented with 2% glycerol, with a working volume of 300 L. A total of 28 vials of overnight-cultured *P. pastoris C1* inoculum, pre‑grown in the same medium, were used for initiation. The bioreactor was maintained at 33 °C and pH 5.6 throughout the process. Agitation began at 100 rpm and was gradually raised to a maximum of 300 rpm based on oxygen demand, while aeration started at 0.55 vvm and was increased up to 1.5 vvm accordingly. Upon depletion of glycerol in the batch medium, a fed‑batch phase was initiated by continuous methanol feeding. Throughout fermentation, residual methanol levels were monitored in real time to dynamically adjust the feed rate, and both biomass accumulation and methanol consumption were recorded at 2‑hour intervals. After fermentation, the entire cell biomass was harvested and subjected to enzymatic hydrolysis. The key enzymatic hydrolysis step was conducted under optimized conditions to maximize peptide yield. Disrupted yeast biomass was sequentially digested with Alcalase (2%, 55°C, pH 8.0, 2 h), Trypsin (1:50, 37°C, pH 8.0, 4 h), and Papain (1.5%, 60°C, pH 6.5, 3 h) to hydrolyze cellular proteins^42^, and enzymes were heat-inactivated (90°C, 10 min) after each digestion step. Critical reaction parameters, including enzyme-to-substrate ratio, temperature, pH, and time, were systematically optimized for each enzymatic stage using response surface methodology to ensure reproducible and composition-directed hydrolysis aimed at yield maximization. The resulting peptides were then separated, washed, dried, and stored at –20°C.

### Nutritional composition analysis

Total protein content was estimated from the total nitrogen content, determined by the Kjeldahl method using a conversion factor of 6.25^43^, and is expressed as a percentage of yeast dry mass. Total peptide content was measured by ultraviolet absorption at 214 nm. Total protein, and peptides content are related to yeast dry mass (%).

Peptide samples were prepared at 1 mg/mL in 0.1% FA and desalted using a column activated with methanol, acetonitrile, 50% acetonitrile, and 0.1% FA. After two loadings and a wash with 0.1% FA, peptides were eluted with 0.1% FA/50% acetonitrile (1:1), lyophilized, and stored at -80°C. For LC-MS/MS analysis, peptides were separated on a 15 cm C18 column ((ReprosilPur 120 C18-ÀQ, 1.9 μm, Dr. Maisch) using a 15-min gradient from 5% to 90% acetonitrile at 600 nL/min and 55°C. MS data were acquired in dia-PASEF mode (m/z 399.6-1199.6; 1/K0 0.67-1.33) with 18×40 Da windows and 120 ms ramp time. Data were processed in Spectronaut (v20.1) via directDIA using the *K. phaffii GS115* database (FDR ≤ 1%). iBAQ values were derived from MS2-level quantification.

Homogenized samples were processed by adding 1 mL of 70% methanol solution and a 3 mm steel ball to solid samples, followed by grinding (JXFSTPRP-48 automated grinder, 70 Hz, 3 min) and vortex mixing (10 min). Liquid samples were mixed with pure methanol and vortexed for 10 min. Sample was then centrifuged (12,000 rpm, 10 min, 4°C), and the supernatant was filtered through a 0.22 μm membrane. An internal standard solution (2-amino-3-(2-chlorophenyl) propanoic acid, 100 μg/mL) was added to a final concentration of 1 mg/L prior to LC-MS analysis. Small-molecule peptides were quantified using LC-MS analysis (Thermo, Ultimate 3000LC, Q Exactive HF).

### Cell lines, culture and functional analyses

Intestinal porcine epithelial cell line-J2 (IPEC-J2) were maintained in 100 mm² uncoated plastic flasks using DMEM supplemented with 10% FBS (Gibco, USA), 5 mM _L-_glutamine, and antibiotic solution (100 U/mL penicillin and 100 μg/mL streptomycin)^44^. Cultures were incubated at 37°C with 5% CO₂ atmosphere, and all experimental procedures utilized cells between passages 10-25.

IPEC-J2 cell proliferation was assessed using CCK8 assay (Beijing solarbio Bioscience & Technology Co., Ltd, Beijing, China). Cells (1.0×10⁴/well) were seeded in 96-well plates, cultured (37°C, 5% CO₂) for 24 h, then treated with PPBP (0, 1, 2, 3, and 4 ug/mL) for 24 hours. After incubation, 10 μL of CCK8 solution was added to each well and incubated at 37 °C for 2 h. A Tecan Infinite M200 PRO multimode reader (Switzerland) was used to measure absorbance at 450 nm, and cell proliferation ratios were calculated according to established methods.

### Molecular docking with PEPT1

According to Killer, PepT1 (ID: 7pmx) was obtained from https://www.rcsb.org/. AutoDock Vina was used for docking between peptides and PepT1. The coordinate information for docking with peptides were x = 102.403, *y* = 95.359, and z = 93.926, and the griddimensions (x, y, z) were 70, 80, and 110, respectively. The binding sites between peptides and PepT1 were analyzed by LigPlot and PyMOL.

### Animal experiments

The animal experiment was approved by the Animal Care Committee of the Institute of Subtropical Agriculture, Chinese Academy of Sciences and was performed in compliance with its guidelines (approval nos. ISA-2022-0060).

Forty pregnant sows (Large White× Landrace) with similar parity (3.35 ± 0.187), backfat thickness (BF, 16.62 ± 0.650) and proximate parturition dates were used in the trial. Throughout the gestation period, sows were housed individually until day 107, after which they were moved to farrowing crates. Post-farrowing, piglets were tagged based on their mothers’ treatments and remained with the sows until weaning at 21 days old.

On day 80 of gestation, the forty sows were randomly divided into two dietary treatment groups: (1) control diet without PpBP (CON; *n* = 20 sows), and (2) control diet with 2 g/kg of PpBP (PpBP; *n* = 20 sows). The randomization was controlled for parity, backfat thickness, and parturition date to ensure balanced groups. The trial lasted from gestation day 85 to lactation day 21, following a 5-day pre-feeding acclimation period. The sows were provided with corn-soybean-based diets, wherein all the essential nutrients were in line with the requirements recommended by NRC (2012) for sows. The protocols for disease prevention, healthcare, and overall management were in strict accordance with the standard practices of the farm. This experiment was conducted at Hebei Rui La Agriculture Co., Ltd. (Langfang, 065601, China). The housing and breeding management procedures were carried out in accordance with the detailed description provided recently^45^.

### Sample collection of sows and neonatal piglets

During the farrowing process, the reproductive performance metrics of sows were meticulously documented. These encompassed the litter size, the quantity of stillbirths and IUGR, and the birth weights of the piglets. IUGR is characterized by the suboptimal growth and development of a fetus during gestation. In the present study, piglets whose birth weights were ~65% of that of the largest littermate within each individual litter were designated as IUGR piglets, following established criteria for the identification and classification of growth-restricted offspring in sows’ studies^46^.

During 24 h post-farrowing, litter size was equalized within treatment to achieve 10 ~ 11 pigs per sow. The number and weight of litter on d1 after cross-fostering and d 20 were recorded; the average daily gain (ADG) and survival rate of piglets were calculated. Also, the ADG in piglets/litter during d1 to 20 were calculated^12^.

On the day of parturition, eight sows per group were randomly chosen for sampling. From each selected sow, a 5 mL blood sample was collected from the jugular vein using a sterile vacuum blood collection tube. In addition, placenta labeling was performed according to the method described by van Rens et al.^47^. Briefly, each delivered piglet was immediately caught, and its umbilical cord was ligated using coded surgical silk. The cord was then severed between the piglet and the tag, allowing the tagged end to retract into the vagina. Piglets were tagged with numbers corresponding to their cord tags. Following expulsion of all placentas in a litter, placentas corresponding to piglets selected based on birth weight and gender within each litter were collected for blood and tissue sampling. Subsequently, 5-mL blood samples were collected from the umbilical veins of eight placentas in each group. Blood samples were centrifuged at 3000 × *g* for 10 minutes at 4 °C to obtain serum, which was then stored at −20 °C for analysis. Moreover, fresh placental samples were collected from vascular‑rich regions ~5 cm from the placental center (umbilical cord), as these areas are considered representative of overall placental status. Approximately 100 g of placental tissue (*n* = 8 per group) was surgically excised and then cut into pieces to ensure their uniformity. These pieces were sub‑packaged and flash‑frozen in liquid nitrogen for subsequent analysis.

On the day of birth, eight neonatal male piglets were randomly selected from CON (1.39 ± 0.063 kg, CV% = 12.91%) and PpBP (1.33 ± 0.069 kg, CV% = 14.82%) groups of the eight pre-marked sows and kept separate from the sows to prevent colostrum ingestion. Anesthesia was induced in pigs by intramuscular injection of a cocktail containing 3% pentobarbital sodium (1 ml/kg, Beijing Daniel Spulber Biotech) and Sumianxin II (0.1 ml/kg, Jilin Huamu Animal Health Products Co., Ltd.). Induction was considered successful upon observation of characteristic behavioral signs such as head slump, markedly reduced spontaneous activity, ataxia, and recumbency, which typically occurred within 3–5 minutes post-injection. Subsequently, 5 mL of blood was aseptically collected from the heart into heparin-free vacuum blood collection tubes. The blood samples were centrifuged at 3000 × *g* for 10 min at 4 °C to obtain serum, which was stored at −20 °C. Additionally, two hepatic samples were collected from each piglet: one sample was stored in liquid nitrogen, and the other was kept at −20 °C for fatty acid (FA) analysis.

### Serum biochemical index analysis

Serum biochemical parameters were measured using an automatic biochemical analyzer (Synchron CX Pro, Beckman Coulter, USA). Specifically, glucose (GLU), total triglycerides (TG), cholesterol (TC), high-density lipoprotein (HDL), and low-density lipoprotein (LDL) were assayed using reagent kits (Roche Diagnostics, China; respective catalog numbers: 04404483, 20767107322, 04718917190, 07528604190, and 07005806190). In addition, nonesterified fatty acid (NEFA) and total bile acids (TBA) were measured with commercial kits (Nanjing Jiancheng, China; catalog numbers: A042-2-1 and E003-1-1) following the manufacturer’s protocols.

### Serum untargeted metabolomics

Serum metabolomics analysis was performed following literature methods^48^. Briefly, 100 μL serum was extracted with 400 μL 50% methanol/acetonitrile containing internal standards (L-leucine-d3 and TMAO-d9), sonicated (10 min), then centrifuged (11,000 × *g*, 4 °C, 15 min). Supernatants were analyzed using a Thermo Vanquish UHPLC-MS system equipped with an ACQUITY BEH Amide column (2.1 × 100 mm, 1.7 μm) coupled to a Q Exactive HFX mass spectrometer. Gradient elution employed (A) 25 mM ammonium acetate/ammonia in water and (B) acetonitrile: 0–0.5 min (95% B), 0.5 ~ 7 min (95 ~ 65% B), 7–8 min (65 ~ 40% B), 8–9 min (40% B), 9–9.1 min (40 ~ 95% B), 9.1–12 min (95% B). The full scan mass spectrum was generated using information-dependent acquisition (IDA) mode within the acquisition software control (Xcalibur, Thermo Fisher Scientific). The electrospray ionization (ESI) source operated under specific conditions: 50 Arb sheath gas flow rate, 10 Arb Aux gas flow rate, 320 °C capillary temperature, 60,000 full MS resolution, 7500 MS/MS resolution, 10/30/60 collision energy in NCE mode, and a 3.5 kV spray voltage (positive mode). Raw data (converted to mzXML via ProteoWizard) were processed using XCMS for peak alignment, then analyzed by OPLS-DA/PCA (SIMCA-P 16.0.2). Significant metabolites (VIP > 1, FC < 0.5 or >2, *P* < 0.05) were visualized via volcano and heatmap plots, with pathway analysis conducted using the Kyoto Encyclopedia of Genes and Genomes (KEGG) database

### Hepatic GLU, TC, TG levels and enzyme activities of neonatal piglets

Hepatic GLU (A154-2-1), TC (A111-1-1), and TG (A110-1-1) levels were measured using commercial kits (Nanjing Jiancheng, Nanjing, China) according to the manufacturer’s instructions. Additionally, the enzymatic activities of phosphoenolpyruvate carboxykinase (PEPCK, BC3315), glucose-6-phosphatase (G6PC, BC3325), citrate synthase (CS, BC1065), hexokinases (HK, BC0745), and ICDH (BC2165), along with hepatic glucose-6-phosphate (G6P, BC3325) level, were determined in the liver tissues of neonatal piglet using commercial kit (Beijing solarbio Bioscience & Technology Co., Ltd, Beijing, China).

### Hepatic medium- and LCFAs of neonatal piglets

Following the method described by Folch et al., hepatic lipids were extracted using chloroform-methanol. Subsequently, the extracted lipids were transmethylated with boron trifluoride (BF3) and methanolic KOH. FA composition was analyzed via gas chromatography (Agilent 6890), and the results were expressed as a percentage of total FAs^49^.

### Transcriptomic analysis of placenta

Based on the criterion of neonatal piglet birth weight (closest to the group mean), a representative subset of four placental samples per group was selected from the total eight for RNA-seq analysis. Placental transcriptome analysis was conducted as previously described^50^. Briefly, total RNA was extracted, and its integrity and quantity were assessed. The initial total RNA for library construction needs to be ≥1 μg. PolyA-tailed mRNA was enriched using oligo (dT) beads, fragmented, and reverse-transcribed into cDNA. The cDNA was end-repaired, A-tailed, and ligated to adapters, followed by size selection (370–420 bp) and PCR amplification. Library quality was verified via Qubit fluorometry, Agilent 2100 bioanalyzer, and qRT-PCR. Qualified libraries were pooled and sequenced on Illumina platforms, generating 150 bp paired-end reads. Reads were aligned to the reference genome using HISAT2, and gene counts were obtained with FeatureCounts. Differential expression analysis ( | log2FC | > 2, adjusted *P* < 0.05) was performed using DESeq2. Functional enrichment of differentially expressed genes (DEGs) was analyzed via GO, KEGG, and Reactome pathways using clusterProfiler.

### Real-time PCR analysis

IPEC-J2 cells in the logarithmic growth phase were seeded into six-well plates and treated with FBS-free DMEM overnight when cells reached 60–70% confluence. Then the cells were incubated with PpBP (3 μg/mL) as the treatment group and bacteria-free PBS as a vehicle-treated group for 24 hours. Cells were washed three times with sterile PBS before RNA extraction. The cells were washed three times with sterile PBS for collection. The mRNA was extracted as previously described^49^. Subsequently, following the manufacturer’s instructions, the relative mRNA expression levels of *GADPH* (reference gene) and target genes were determined by real-time PCR using Luminaris Color HiGreen High ROX (Thermo Scientific) and a Bio-Rad iCycler. The 2^–ΔΔCt^ method was used to calculate the fold change in mRNA expression. Primer sequences are presented in Supplementary Fig. 1.

### Western blotting

IPEC-J2 cells were seeded in 100 mm² plates and serum-starved in FBS-free DMEM overnight upon reaching 60–70% confluence. The cells were then treated for 24 hours with either 3 µg/mL PpBP. Subsequently, the cells were collected, washed twice with PBS, and processed for protein analysis. Total protein extraction and western blotting were conducted as previously reported^45^. Proteins were separated via 10% SDS-PAGE and transferred to PVDF membranes (Millipore, Billerica, MA) using a Bio-Rad transblot apparatus (Hercules, CA) for 2 hours. Membranes were blocked with 5% fat-free milk in TBST for 3 hours, then incubated overnight at 4 °C with primary antibodies GAPDH (AC002, ABclonal Technology, 1:10000), AKT (9272, Cell Signaling Technology, 1:1000), Phospho-AKT (9271, Cell Signaling Technology, 1:1000), Phospho-p38 MAPK (AP0057, ABclonal Technology, 1:1000), Phospho-PI3K (AP0427, ABclonal Technology, 1:1000), PEPT1 (PA537010, Thermo Fisher Scientific, 1:1000). After washed three times with TBST, the membranes were incubated with horseradish peroxidase-linked secondary antibodies (Beijing ZhongShan Golden Bridge Biological Technology Co., Ltd., China) for 2 hours at room temperature. Following TBST washes, membranes were developed using Supersignal West Dura substrate (Pierce, Rockford, IL). The images were detected by chemiluminescence (Applygen Technologies Inc., Beijing, China), and the images were quantified by measuring the intensity of correctly sized. Band intensities were quantified densitometrically using ImageJ software (National Institutes of Health, USA). For each sample, the optical density of the target protein band was normalized to that of GAPDH from the same lane. Normalized values from PpBP-treated groups were then expressed relative to the mean of the control group (set as 1.0) to obtain a fold-change in protein expression.

### Statistical analysis

Data were analyzed with SPSS 21.0 (IBM-SPSS Inc., Chicago, IL, USA). Normality and homoscedasticity of the data variances were checked using Shapiro Wilk and Levene’s test, respectively. Reproductive performance data were subjected to two-way ANOVA to evaluate the effects of diet, backfat thickness, and their interaction. Other variables (e.g., individual piglet weight, blood parameters) were compared using independent-samples *t* tests. The data were presented as the mean values. Probability values < 0.05 and <0.01 were considered statistically significant and extremely significant, respectively.

## Supplementary information

Supplementary material

## Data Availability

The raw RNA sequencing data generated in this study have been deposited in the NCBI BioProject database under accession number PRJNA1418818 (project link: https://www.ncbi.nlm.nih.gov/bioproject/1418818). All other relevant data supporting the findings of this study are available within the manuscript and supplementary data.
