# Supplementary material for: One-carbon-derived bioactive peptides improve reproductive performance via regulating placental nutrient transport and offspring glycolipid metabolism
Source: NPJ Sci Food. 2026 Feb 27;10:120. doi: 10.1038/s41538-026-00769-9 (PMC13066491; doi:10.1038/s41538-026-00769-9)

Supplementary Table 1: Primers used for real-time PCR

Genes	Accession No.	Nucleotide sequence of primers (5'-3')
PEPT1	NM_214347.1	F: TTCCTTCAGGCGCAAAAAGC<br>R: CACATAGGTAAACGCGCTGC
PEPT2	NM_001145998.2	F: CTGCTGTGGTCATGGCTGAAG<br>R: GGTACGTCACCTGGAAAGTGG
SNAT1	XM_003355629.4	F: AAGAACCTGGGCTATCTCGG<br>R: TGTTGCGTTAGGACTCGTTG
SNAT2	XM_013997964.2	F: TACTTGGTTCTGCTGGTGTCC<br>R: GTTGTGGGCTGTGTAAAGGTG
LAT1	NM_001110421.1	F: TTTGTTATGCGGAACTGG<br>R: AAAGGTGATGGCAATGAC
EAAT1	NM_001244425.2	F: GATGGGACCGCCCTCTAT<br>R: CGTGGCTGTGATGCTGATG
ASCT2	XM_003355984.4	F: GATTGTGGAGATGGAGGATGTGG<br>R: GCGAGTGAAGAGGAAGTAGATGA
PAT1	XM_021077073.1	F: TGTGGACTTCTTCCTGATTGTC<br>R: CATTGTTGTGGCAGTTATTGGT
HK1	NM_001243184.1	F: GCTCACCAGAGGGAAGTTCAACAC<br>R: CCAGGCGGGTCAGGATTTCTTTG
G6PC	NP_001106916.1	F: AAGCCAAGCGAAGGTGTGAGC<br>R: GGAACGGGAACCACTTGCTGAG
pFkm	NM_001044550.1	F: TTAGCGGCAGCTTTGATAGTTC<br>R: CACAATGACCACATCTCCCTTC
PCK1	NM_001123158.1	F: TCAGCACGACTCCAGCCTTCA<br>R: GCTCAAGCAGTCTGGGCATTCT
PCK2	NM_001161753.1	F: ACAGGAGGTTCGTGACATTCGG<br>R: GTGGTGCTGTGCTCACTTGCTA
FBP1	NM_213979.1	F: TCCACCGCACGCTGGTCTAT<br>R: CCAGTCCTCCTGCCTTCTCCAT
PC	NM_214349.1	F: CCGCAAGATGGGAGACAAGGT<br>R: GGAAGCCGATGGTGTTGGAGAA
CS	NM_214276.1	F: TCTCAGCTCAGTGCAGCCATTACA<br>R: CTGCAACACAAGGTAGCTTTGCGA
OGDC	XM_021079061.1	F: GCGGTCCACCAGGTTTGAAGAG<br>R: CCACTCCGTTCTCACTTGACTTGTC
ICDH-γ	XM_021079674.1	F: TGTGGTTCCTGGTGAGAG<br>R: CGAGATTGAGATGCCGTAG
ICDH-β	XM_047771459.1	F: GGTGGAGAGCCTCAAGAT<br>R: TGGTGGTGTTGTCTACGA
GLUT-2	NM_001097417.1	F: GGTTCATGGTGGCCGAGTT<br>R: ATTGCGGGTCCAGTTGC
SGLT-1	NM_001012297.1	F: TCATCATCGTCCTGGTCGTCTC<br>R: CTTCTGGGGCTTCTTGAATGTC

FAT/CD36	XM_021102279.1	F:CTGGTGCTGTCATTGGAGCAGT<br>R:CTGTCTGTAAACTTCCGTGCCTGTT
FABPpm	NM_213928.1	F:ATGGGCTTATACGGTGAGCG<br>R:CGTTGACAGGAGGGTTGGAA
FATP1	XM_021076151.1	F: GGAGTAGAGGGCAAAGCAGG<br>R: AGGTCTGGCGTGGGTCAAAG
FABP2	NM_001031780.1	F: CGGAACTGAACTCACTGGGAA<br>R: CTGGACCATTTCATCCCCGA
ACC2	XM_021066238.1	F: GCCGAAACATCTCTGGGATA<br>R: CTCCAGGACAGCACAGATCA
CPT1-α	NM_001129805.1	F: CCATCAAAACTGCCTTCCTTAG<br>R: AGCGAGTGTGCCAGATACAAA
CPT1-β	NM_001044575.2	F: TTCCGCCAAACCTTGAAACT<br>R: GGACACAGATAGCCCAGACTTT
Cyp7-α	XM_013996745.2	F: ACCTGACCAGTTCCGAGATG<br>R: TATAGGGCACGATGCACAGA
SCD	NM_213781.1	F: AAGGAGCTGGTCAGTCGTTG<br>R: GCTTTCGAAGCTTTGTGCCA
ATGL	NM_001098605.1	F: GACCTGATGACCACCCTTTC<br>R: CAGATACTGGCAGATGCTACC
ELOVL-2	XM_021100180.1	F: ATTCTTCACCACCAGCGAGG<br>R: TGCCTGGCTGTTATCACTCG
ELOVL-5	XM_021098832.1	F: TACCACCATGCCACTATGCT<br>R: GACGTGGATGAAGCTGTTGA
FADS1	NM_001113041.1	F: GTCACTGCCTGGCTCATTCT<br>R: AGGTGGTTCCACGTAGAGGT
HSL	XM_021093574.1	F: GAGGGTTCAAGAAAGGCCCA<br>R: TACCCGCAAAGACACCTGAC
SREBP1	NM_214157.1	F: TCTGGAGACATCGCAAACAAG<br>R: AGGTGGCGGATGAGGTTC
BCL2L-1	NM_214285.1	F: GGTACCGGAGGGCATTCAG<br>R: ACAATGCGACCCCAGTTCAC
BCL2	XM_021099593.1	F: AGGATTGTGGCCTTCTTTGAGTT<br>R: CGGTTCAGGTACTCAGTCATCCA
FOS	NM_001123113.1	F: GAGCTGACTGACACACTCCA<br>R: GCGATCTCAGTCTGCAAAGC
MYC	NM_001005154.1	F: CGGACACGGAGGAGAATGAC<br>R: GCTGCGTTTCAGCTCGTTTC
P53	NM_213824.3	F: CTGCTTCCTGAAAACAACC<br>R: AAGGGACAAAGGACGACA
RASFF4	XM_071196413.1	F: AGCTGATGCGGACTAAGAGC<br>R: CACGGAGGTCTTGTGGTTGT
WNT16	XM_003134736.4	F: AGTGTTCCTGTGACACCACC<br>R: TTCTGCTGAACCACATGCCA

PDGFRA	NM_001315756.1	F: CCTACATCGGCGTCACCTAC<br>R: GGCAGAGGGATGATGTAGCC

Supplementary Figure 1: GADPH expression in IPEC-J2 cells

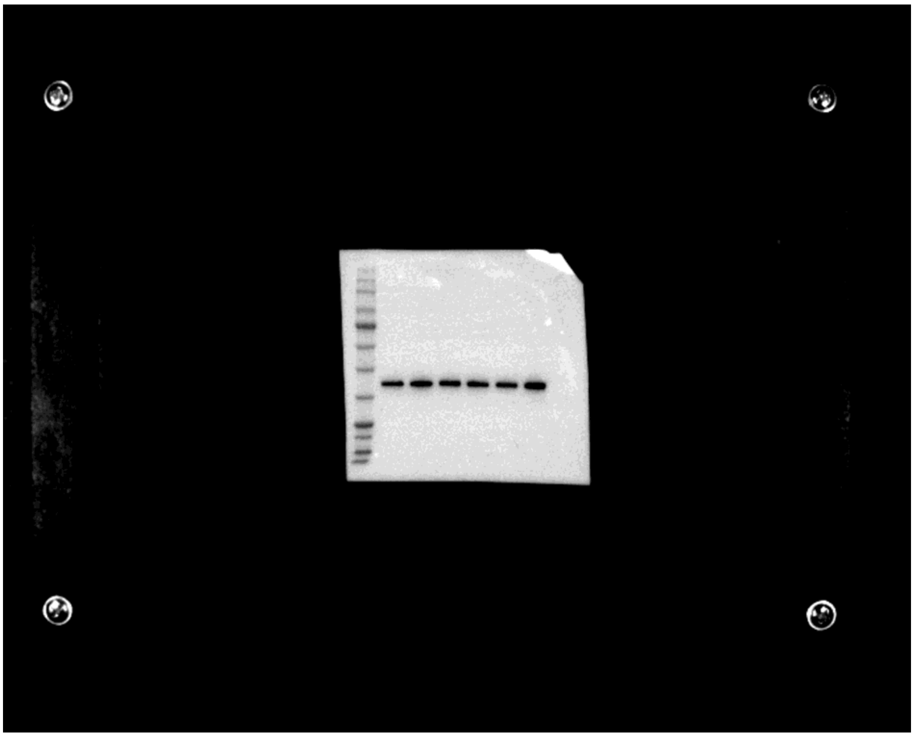

Supplementary Figure 2: PEPT1 expression in IPEC-J2 cells

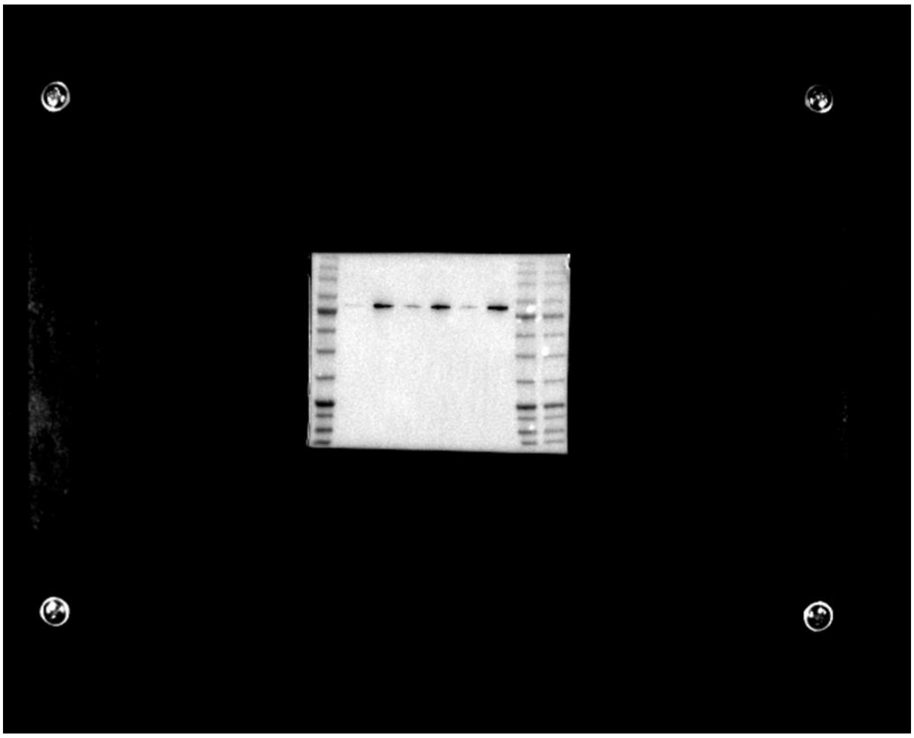

Supplementary Figure 3: GADPH expression in placenta

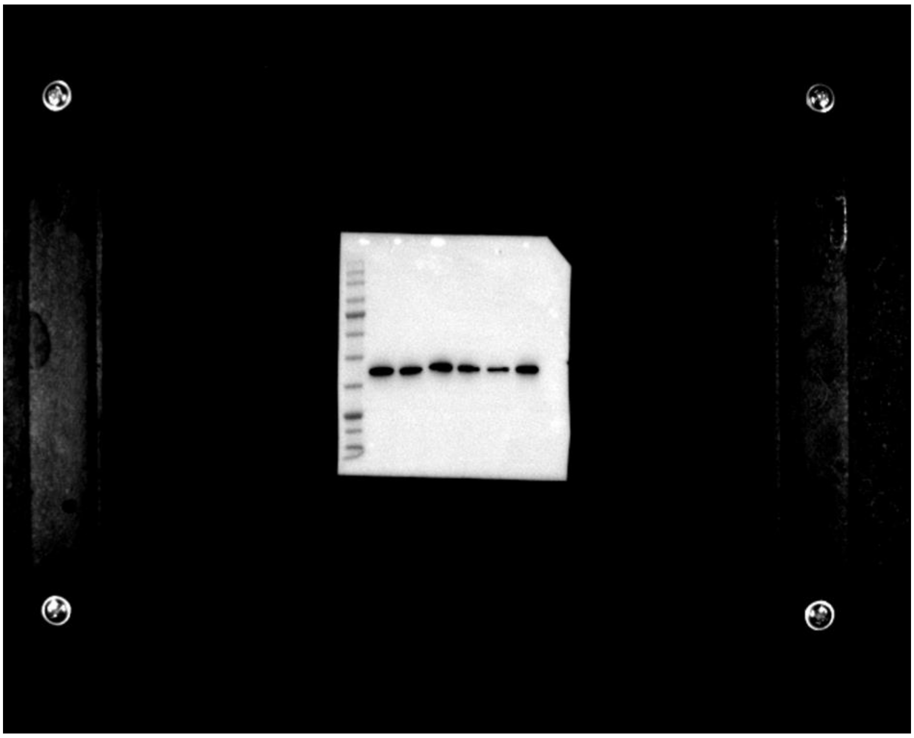

Supplementary Figure 4: P38MAPK expression in placenta

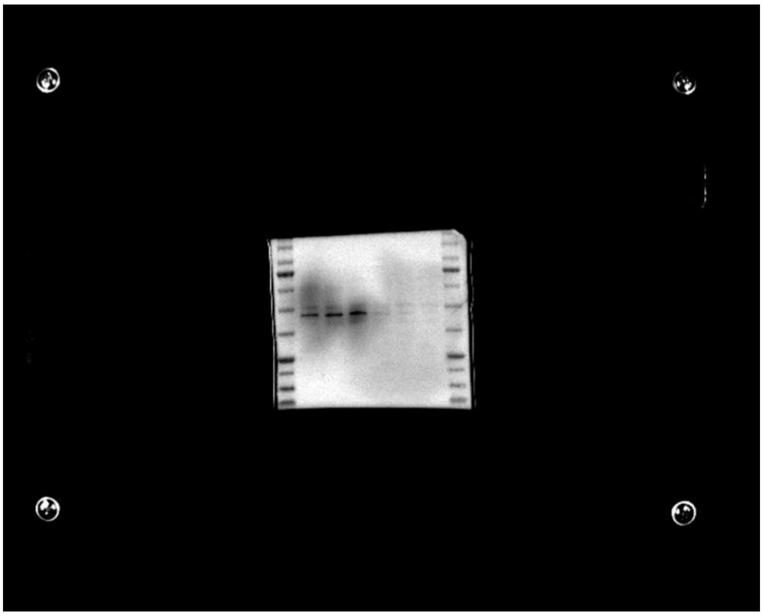

Supplementary Figure 5: P-PI3K expression in placenta

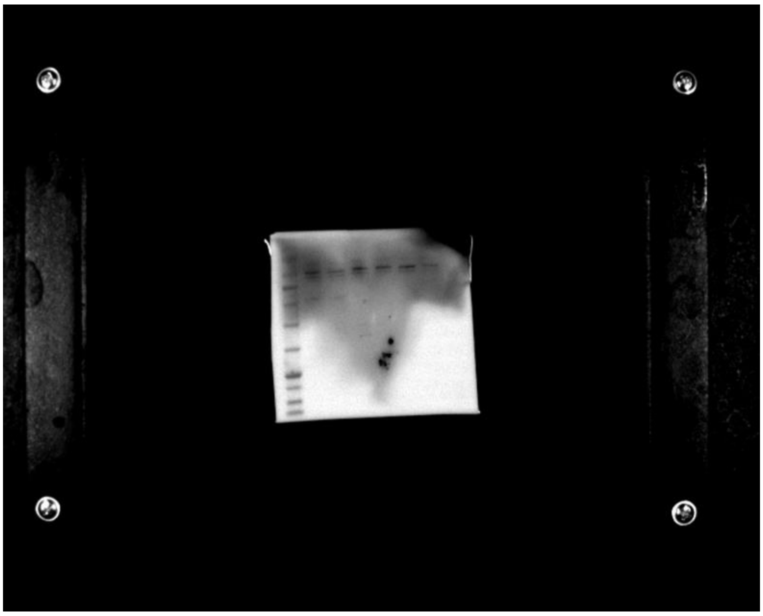

Supplementary Figure 6: AKT expression in placenta

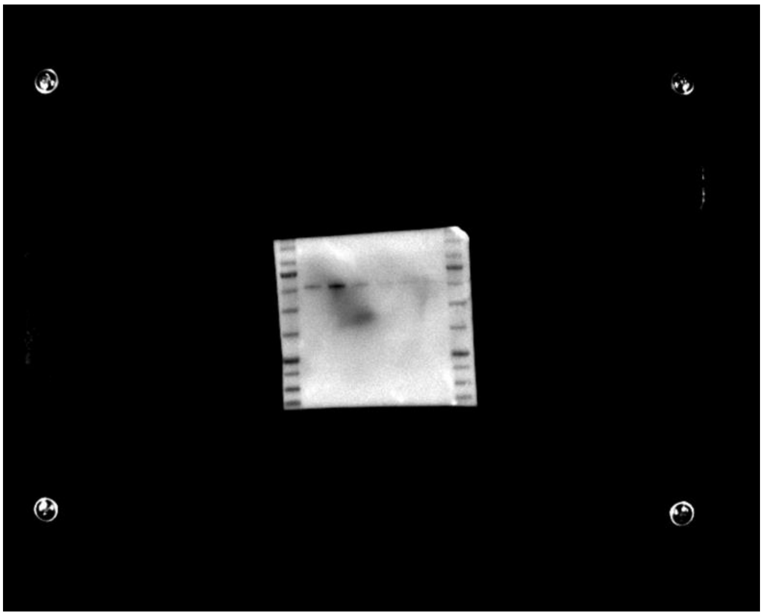

Supplementary Figure 7: P-AKT expression in placenta

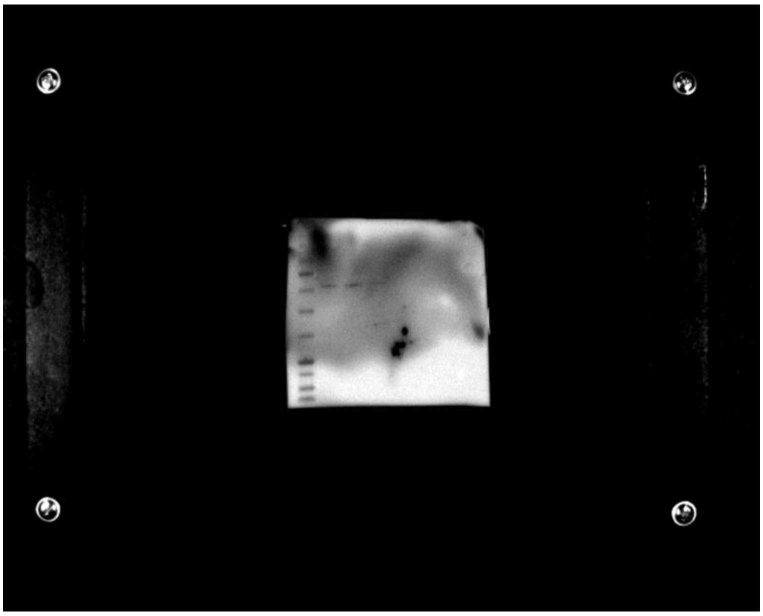

Supplement: Supplementary file 1 — Supplementary material [file 41538_2026_769_MOESM1_ESM.pdf]
